# Desulfurization of Dibenzothiophene and Oxidized Dibenzothiophene Ring Systems

**DOI:** 10.3390/molecules15031265

**Published:** 2010-03-03

**Authors:** Diego P. Morales, Alexander S. Taylor, Steven C. Farmer

**Affiliations:** Department of Chemistry, Sonoma State University, 1801 East Cotati Avenue, Rohnert Park, CA 94928, USA

**Keywords:** dibenzothiophene, desulfurization, sulfoxides, sulfones

## Abstract

Lithium, used in conjunction with sodium metal, produces a high yield of biphenyl when reacted with dibenzothiophene, dibenzothiophene sulfoxide or dibenzothiophene sulfone.

## 1. Introduction

The reductive desulfurizations of various types of organosulfur compounds are of importance in industry, mainly because of their application in the desulfurization of fossil fuels to reduce pollution [1,2]. The desulfurization of polycyclic aromatic sulfur compounds such as benzo- and dibenzothiophenes are of particular interest because of their widespread occurrence in fossil fuels. 

The desulfurization of polycyclic aromatic sulfur compounds also has applications in the laboratory [3,4,5]. Because the sulfur atom in such heterocycles has a strong directing effect towards electrophillic aromatic substitution reactions, the subsequent desulfurization could provide a regioselective entry to substituted biphenyls and related compounds. Because of this and the fact that dibenzothiophenes are particularly difficult to desulfurize by conventional methods, new methodology that can efficiently perform this reaction are of great interest. Examination of the literature dealing with the desulfurization of dibenzothiophene shows that numerous synthetic problems remain unsolved and that room for new reagents exists. Some examples of the desulfurization of dibenzothiophene (**1**) to make biphenyl (**2**) have been reported, however these reactions involve long reaction times [6,7], high temperatures [8,9,10], complex reagents [11] or low yields [16]. In addition there have been very few examples of the desulfurization of dibenzothiophene sulfoxide (**3**) [17,18], and dibenzothiophene sulfone (**4**) [10,19,20,21] as *S*-oxidized derivatives of this ring system. In fact, both lithium and sodium [10] have been used to effect sulfur extrusions from dibenzothiophene. However, in both cases low yields occurred unless high temperatures were used. The high temperatures required that the high boiling point solvent, tetradecane, be used. The removal of high boiling point solvents from reaction mixtures can be difficult. The generation of a method which can afford high sulfur extrusion yields, while still using a solvent which can be removed by rotary evaporation, would be of great benefit. In this manuscript we apply our previous work using lithium in combination with sodium as an efficient method for the desulfurization of heterocycles [22,23]. 

## 2. Results and Discussion

**Scheme 1:**
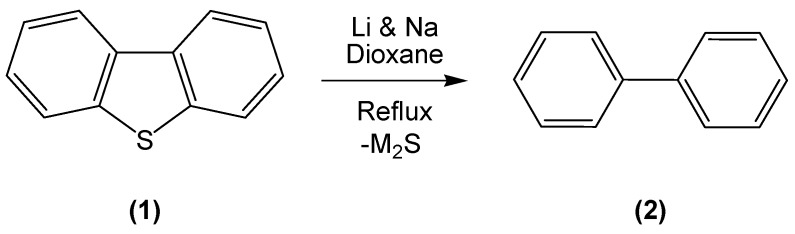
The desulfurization of dibenzothiophene (**1**).

**Scheme 2:**
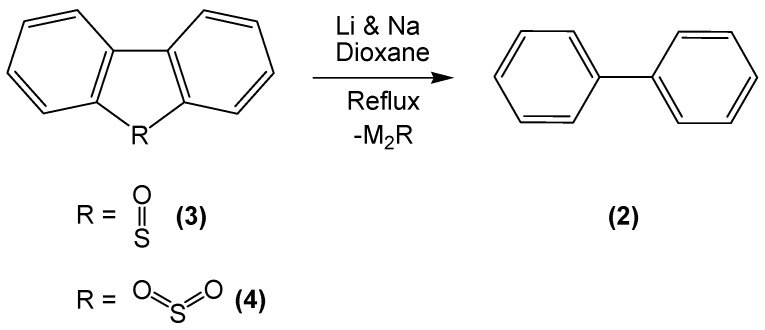
The desulfurization of dibenzothiophene sulfoxide (**3**) and sulfone (**4**).

In the past we have increased the sulfur extrusion ability of lithium by using it in conjunction with sodium. By combining these two metals we were able to utilize the increased reducing ability of sodium while still maintaining the sulfur removing effects of lithium. Also, the synergistic effect of using both metals together allowed for the reaction temperature to be reduced such that refluxing dioxane could be used. This solvent was easily removed by rotary evaporation during the reaction work-up. The use of sodium in conjunction with lithium increased the yield of biphenyl from dibenzothiophene to 88% (Scheme 1). Owing to the dianion intermediate mechanism proposed by Gilman [16] it was decided that the sulfoxide and sulfone derivatives of dibenzothiophene could stabilize the intermediates of this reaction; thereby, increasing the biphenyl yield (Scheme 2). This turned out to be the case with dibenzothiophene sulfoxide, which produced a biphenyl yield of 90%. Dibenzothiophene sulfone also produced an increased biphenyl yield (96%). In all cases the reactions produced no side products. Occasionally, some starting material would be isolated. 

## 3. Experimental

### 3.1. General

All infrared spectra were recorded on a Perkin Elmer Spectrum 1000 FTIR infrared spectrometer and were performed neat on a NaCl plate. ^1^H-NMR were obtained on a General Electric QE-300 at 300 MHz in CDCl_3_ using TMS as the reference. ^13^C-NMR were obtained at 75 MHz in CDCl_3_. All GCMS spectra were recorded using a Shimadzu GCMS-QP500 gas chromatography/mass spectrometer. All melting points are uncorrected and were measured using a Mel Temp 3.0 melting point apparatus. Chromatography was accomplished using 20 to 230 mesh silica gel. Thin layer chromatography was performed using plastic backed silicagel 60 F_254_ plates. Unless otherwise stated, all reagents were obtained from commercial sources and were used without further purification. The compound dibenzothiophene sulfoxide (**3**) was made using a literature method [24].

### 3.2. Biphenyl *(**2**)* from dibenzothiophene *(**1**)* using Li/Na

In a 25 mL round-bottomed flask dibenzothiophene (0.150 g, 0.814 mmol) was dissolved in dioxane (10 mL). To this was added lithium dispersion (0.050 g, 7.20 mmol) in mineral oil and sodium metal (0.200 g, 8.70 mmol). The mixture was heated at reflux for 12 h, after which time any remaining lithium or sodium was destroyed with careful addition of methanol. The organic solvent was removed by evaporation and the residue was dissolved in water (20 mL) and ethyl acetate (20 mL). The layers were separated and the aqueous layer was extracted two more times with ethyl acetate (20 mL). The combined organic layers were dried with sodium sulfate, filtered, and evaporated to produce a white powder. Purification consisted of silica column chromatography, using hexane as the elution solvent. After work-up the reaction produced 0.110 g (0.713 mmol) of pure biphenyl, a yield of 88%. m.p. 68.4–69.1 ºC (lit. m.p. 68–69 ºC [25]); IR (cm^−1^, Neat): 3,033 (w, Ar-H), 1,482 (w, C=C), 1,430 (w, C=C); ^1^H-NMR: δ (ppm) 7.58 (d, *J* = 7.5 Hz, 4H), 7.42 (t, *J* = 7.5 Hz, 4H), 7.33 (t, *J* = 7.5 Hz, 2H); ^13^C-NMR: δ (ppm) 141.21, 128.73, 127.23, 127.15 [25]; MS (EI, *m/z*) 154 (M^+^).

### 3.3. Biphenyl *(**2**)* from dibenzothiophene sulfoxide *(**3**)* using Li/Na

The same general procedure was followed as above except sodium metal (90.0 mg, 3.91 mmol) and lithium dispersion (60.0 mg, 8.64 mmol) was reacted with dibenzothiophene sulfoxide (0.100 g, 0.499 mmol). The reaction was refluxed for 24 h. The reaction produced 69.2 mg (0.449 mmol) of pure biphenyl, a yield of 90%. m.p. 69.3–71.7 ºC; IR, NMR and MS, identical in all respects to an authentic sample. 

### 3.4. Biphenyl *(**2**)* from dibenzothiophene sulfone *(**4**)* using Li/Na

The same general procedure was followed as above except sodium metal (0.100 g, 4.35 mmol) and lithium dispersion (60.0 mg, 8.64 mmol) was reacted with dibenzothiophene sulfone (0.100 g, 0.462 mmol). The reaction was refluxed for 24 h. The reaction produced 68.3 mg (0.443 mmol) of pure biphenyl, a yield of 96%. m.p. 69.3–70.5 ºC; IR, NMR and MS, identical in all respects to an authentic sample. 

## 4. Conclusions

By providing an efficient, high yield method for the desulfurization of dibenzothiophene and *S*-oxidized dibenzothiophene ring systems, we have opened more possible synthesis routes for biphenyl derivatives. These improvements may now be applied to other types of compounds that contain the thiophene ring system.
